# Colostomy May Improve Outcomes in Patients with Severe Anogenital and Gluteal Hidradenitis Suppurativa: Case Series

**DOI:** 10.2340/actadv.v106.adv-2026-0533

**Published:** 2026-08-25

**Authors:** Valdis Thorhallsdottir, Karin Berggård, Meirav Holmdahl, Martin Lindsten, Louis Banka Johnson, Artur Schmidtchen, Anja Pahlow Mose

**Affiliations:** 1 Division of Dermatology and Venereology, Department of Clinical Sciences Lund, Lund University, Lund, Sweden; 2 Department of Dermatology, Skane University Hospital, Lund, Sweden; 3 Division of Surgery, Department of Clinical Sciences Malmö, Lund University, Malmö, Sweden; 4 Department of Surgery, Pelvic Floor Centre, Skane University Hospital, Lund, Sweden

Hidradenitis suppurativa (HS) is a painful chronic inflammatory skin disease with an estimated prevalence of 1% (1). It is characterized by recurrent inflamed nodules, abscesses and leaking tunnels in the apocrine gland bearing regions of the body. The lesions often cause great pain, malodorous discharge and irreversible scarring leading to augmented inflammation and even more scarring. HS imposes a substantial disease burden, with patients frequently reporting marked impairment in quality of life (2). HS has a female predominance (3 : 1) but an increased disease severity in men with greater gluteal involvement (3). Despite recent advances, the pathogenesis of HS is still poorly understood and so far, treatment remains a challenge.

Recent studies suggest a multifactorial pathogenesis including both genetic and environmental factors where an altered skin and gut microbiota together with dysregulation of the local immune system appears to be important (4, 5). The interaction between the microbiome and the human host is thought to be an important factor for maintaining normal homeostasis. Changes in the microbiome could result in dysbiosis, leading to immune system reactivity and possibly inflammatory disease development such as in HS (6, 7). However, the exact mechanisms by which the skin and gut microbiomes may contribute to the development of HS still remain unclear (4).

Here, we present a case series of 4 patients from our cohort that have received colostomy as a treatment for severe debilitating anogenital/gluteal HS, initially for wound management purposes prior to planned extensive surgery.

## CASE SERIES

Four patients with severe debilitating anogenital HS (Hurley grade III) diagnosed by a specialist dermatologist received colostomy in preparation for extensive surgery. The demographics, medical and treatment history of each patient are outlined in **Table I**. All 4 patients had received treatment with multiple biological medications for several years prior to colostomy. Three months before surgery, consistent with a state of chronic inflammation, all patients had elevated CRP and 3 of 4 had haemoglobin (Hb) levels under the lower reference value. Of special note, patient 1 was treated with adalimumab+methotrexate under a 7-month period 3 years before receiving colostomy with insufficient effect. Eight weeks post-operatively the patient started treatment again with adalimumab+methotrexate. Patient 2 had multiple severe flare-ups with CRP >100 mg/L before receiving colostomy. At 6-months pre-op, the patient had a flare-up that was treated with peroral antibiotics. Patient 3 switched from infliximab to ustekinumab 3 months after receiving colostomy because of neutralizing antibodies against infliximab. At that time, CRP levels had decreased from 51 mg/L to 17 mg/L.

**Table I.** Patient characteristics

Patient	1	2	3	4
Sex	F	M	M	M
Age	62	57	34	59
BMI pre-op	32	28	27	31
Medical history	Arthritis	Asthma Depression Nephrolithiasis Previous alcoholism	Primary biliary cholangitis	High blood pressure Sleep apnoea
History of smoking	No	Yes	Yes	Yes
Baseline hurley	III	III	III	III
Systemic treatments for HS before colostomy	Clindamycin Rimactan Acitretin Infliximab Adalimumab Methotrexate Ustekinumab	Tetracycline Adalimumab Infliximab Ustekinumab	Isotretinoin Prednisolon Tetracycline Adalimumab Infliximab Ustekinumab	Adalimumab Infliximab Ustekinumab
Systemic treatment at time of colostomy	Secukinumab	Ustekinumab	Infliximab	Bimekizumab
Systemic treatment 12 months after colostomy	Adalimumab+ Methotrexate	Ustekinumab	Ustekinumab	Bimekizumab

**Fig. 1.**
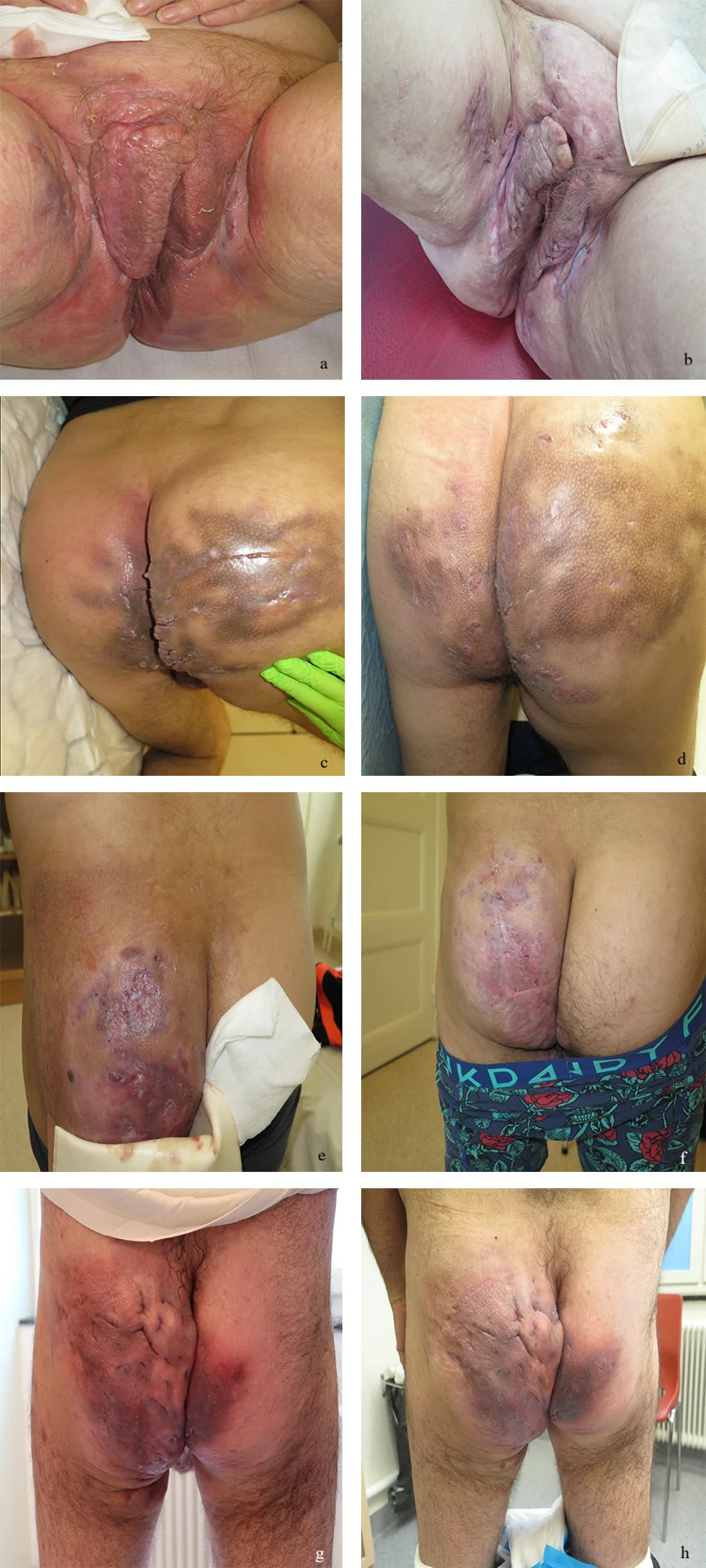
Clinical photographs pre- and post-operatively. (a) Patient I 1-month pre-op (b) patient I 12-month post-op (c) patient II 1-month pre-op (d) patient II 12-month post-op (e) patient III 1-month pre-op (f) patient III 12-month post-op (g) patient IV 1-month pre-op (h) patient IV 13-month post-op] (all abbreviations explained first time mentioned).

**Fig. 2.**
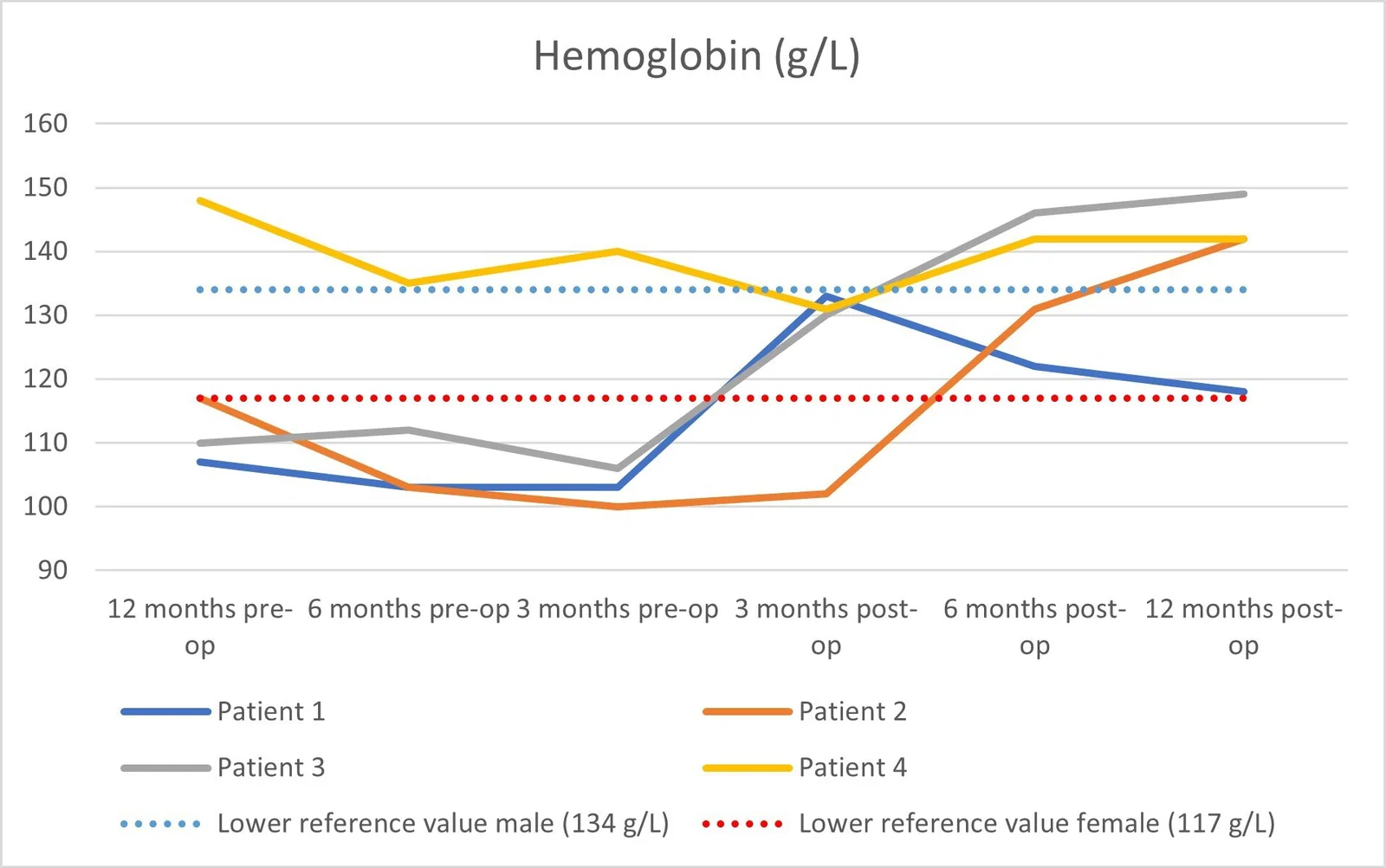
CRP and Hemoglobin in serum pre- and post-operatively.

Within a year after receiving their colostomy the patients experienced reduced secretion from tunnels. The lesional skin was less inflamed and the chronic pain had eased (**Fig. 1**). Blood tests a year after surgery showed a marked decrease in CRP, and Hb was over the reference value in all 4 patients (**Fig. 2**). As of today, none of the patients have wished to follow through with the previously planned extensive surgery due to the marked improvement of their HS.

## DISCUSSION

Receiving colostomy greatly improved the disease course for our patients with HS. Their clinical improvement is validated by the distinct improvements seen in their inflammatory markers and improvement in Hb levels. Further this improvement was maintained 12 months post-operatively. As of today, 2 of 4 patients still have the same biological treatment they had before colostomy. The other 2 switched treatment to a previously used biologic within 3 months of receiving colostomy. These treatments had not been successful for the patients before but in combination with colostomy there was an observed improvement.

Research has shown that the skin microbiota takes part in controlling host physiology, and when uncontrolled, it can promote inflammation. There is increasing evidence that dysbiosis in the skin microbiome can be a contributing factor to pathophysiology of other dermatological diseases such as acne, eczema and psoriasis (8). Taken together, these observations led us to hypothesize whether colostomy can alter the skin microbiota in the anogenital area promoting homeostasis and reducing inflammation.

The skin and gut microbiomes communicate through the gut–skin axis, where changes in one can influence the health of the other (9). HS shares many clinical manifestations, genetic susceptibilities and immunologic features with inflammatory bowel disease, particularly Crohn disease (CD) (10). Further, studies have shown that patients with CD have a 9-fold risk of developing HS compared to the general population (11). Patients with coexisting CD and HS are more likely to have severe HS, have higher rates of anogenital disease and have increased requirement for surgical intervention and probability of a permanent stoma because of their CD (12). Previous research has shown that patients with CD have changes in microbial composition and reduction in species diversity compared to healthy individuals, producing a pathological immune response (13). Stomas are widely used for fecal diversion in patients with CD, and studies have demonstrated that it is an effective treatment option in refractory perianal CD (14).

Colostomies have thus far only been used temporarily in patients with severe gluteal and anogenital involvement prior to elective extensive surgery given the increased risk of surgical site infection and have not played an important role in managing HS (15). In light of our findings, further research is needed if fecal diversion with colostomy is a feasible treatment option for selected patients with severe refractory HS in the anogenital/gluteal area. In addition, our findings raise interesting questions regarding the pathogenesis of HS, the role of the skin–gut axis and the possible shared underlying immunopathogenic mechanisms between HS and CD, where further research is needed.

## Data Availability

The data that support the findings of this study are available from the corresponding author upon reasonable request.
